# The cost-effectiveness of upfront point-of-care testing in the emergency department: a secondary analysis of a randomised, controlled trial

**DOI:** 10.1186/s13049-019-0687-2

**Published:** 2019-12-11

**Authors:** Lara Nicole Goldstein, Mike Wells, Craig Vincent-Lambert

**Affiliations:** 1 Division of Emergency Medicine, Faculty of Health Sciences, University of the Witwatersrand, Johannesburg, South Africa; 2 Department of Emergency Medical Care, Faculty of Health Sciences, University of Johannesburg, Johannesburg, South Africa

**Keywords:** Point-of-care systems, Point-of-care testing, Emergency department, Economic analysis

## Abstract

**Background:**

Time-saving is constantly sought after in the Emergency Department (ED), and Point-of-Care (POC) testing has been shown to be an effective time-saving intervention. However, when direct costs are compared, these tests commonly appear to be cost-prohibitive. Economic viability may become apparent when the time-saving is translated into financial benefits from staffing, time- and cost-saving. The purpose of this study was to evaluate the cost-effectiveness of diagnostic investigations utilised prior to medical contact for ED patients with common medical complaints.

**Methods:**

This was a secondary analysis of data from a prospective, randomised, controlled trial in order to assess the cost-effectiveness of upfront, POC testing. Eleven combinations of POC equivalents of commonly-used special investigations (blood tests (i-STAT and complete blood count (CBC)), electrocardiograms (ECGs) and x-rays (LODOX® (Low Dose X-ray)) were evaluated compared to the standard ED pathway with traditional diagnostic tests. The economic viability of each permutation was assessed using the Incremental Cost Effectiveness Ratio and Cost-Effectiveness Acceptability Curves. Expenses related to the POC test implementation were compared to the control group while taking staffing costs and time-saving into account.

**Results:**

There were 897 medical patients randomised to receive various combinations of POC tests. The most cost-effective combination was the i-STAT+CBC permutation which, based on the time saving, would ultimately save money if implemented. All LODOX®-containing permutations were costlier but still saved time. Non-LODOX® permutations were virtually 100% cost-effective if an additional cost of US$50 per patient was considered acceptable. Higher staffing costs would make using POC testing even more economical.

**Conclusions:**

In certain combinations, upfront, POC testing is more cost-effective than standard diagnostic testing for common ED undifferentiated medical presentations – the most economical POC test combination being the i-STAT + CBC. Upfront POC testing in the ED has the potential to not only save time but also to save money.

**Trial registration:**

ClinicalTrials.gov: NCT03102216.

## Introduction

Point-of-Care (POC) tests – diagnostic tests that are performed at or near the patient’s bedside – have been touted as potential time-saving interventions to decrease waiting times in the Emergency Department (ED) [1–3]. These tests can decrease the turnaround time of special investigations thereby reducing delays which can cause prolonged patient times in the ED [2, 4]. While these POC time-savers are mostly reported in the literature as being cost-prohibitive to implement when their direct costs are compared to the traditional diagnostic testing, the POC system costs have conversely also been reported as being less expensive than central laboratory costs in other studies [2, 5–7]. Recouping the personnel costs from the time that is saved, however, may paradoxically mean that the more expensive POC tests have financial benefit and therefore become an economically viable option [2, 5]. The improved overall processing of the patient as a result of the reduced turnaround times, more rapid diagnosis and disposition could potentially allow for fewer staff members to manage the same number of patients in the same time as using a conventional system [2, 5, 8]. This would be an important potential consideration when planning and optimising ED staffing.

Conventionally, special investigations such as blood tests, electrocardiograms (ECGs) and radiological investigations take place after an ED doctor has evaluated a patient. Both the patient and the doctor then need to wait for the results of these tests before the doctor can make a disposition decision for the patient. When conventional testing is replaced with POC tests performed upfront prior to doctor assessment, significant time-saving has been demonstrated – this was our randomised, controlled trial that provided the data on which this secondary analysis is based [9]. Whether the time-saving from this intervention could translate into money-saving is important to determine. This information would be useful for policy- and decision-makers with regards to deciding whether to implement upfront, POC testing in their EDs.

The aim of this study was to evaluate the cost-effectiveness of common diagnostic investigations, in the form of POC tests, performed prior to doctor assessment, for patients presenting to the ED as a secondary analysis of data obtained from our randomised controlled trial [9].

## Methods

### Study design and setting

This was a secondary analysis of data from an investigator-initiated prospective, randomised, controlled trial. The original trial evaluated the time-saving potential of upfront, POC tests in the ED [9]. This secondary analysis was conducted in order to assess the cost-effectiveness of the upfront POC testing. The trial was conducted in the ED of a tertiary, academic hospital in a metropolitan area of Johannesburg, South Africa. The ED sees approximately 65,000 patients annually. The hospital is a government-funded, public sector hospital serving a region with a population of approximately one million medium- to low-income people.

The study took place between 13 February and 29 June 2017.

Permission to conduct the study was granted by the Research Ethics Committee of the Faculty of Health Sciences of the University of Johannesburg (REC-01-185-2016); the Human Research Ethics Committee of the University of the Witwatersrand (M171086); South African National Health Research Ethics Council (DOH-27-0117-5628); and was registered as a clinical trial with the South African National Health Research Database (GP_2017RP57_655) as well as with clinicaltrials.gov (NCT03102216). Written informed consent was obtained from all patients. Patients were not paid for their participation in this study nor did they incur any expenses related to the study.

### Selection of participants

During weekdays, all adult patients older than 18 years who presented to the ED with various common medical symptoms were eligible for inclusion in the study. The medical symptom groups included were typical of the so-called undifferentiated patient that may present to the ED viz.

- “abdominal group” – patients who presented with any form of abdominal pain and/or vomiting
- “chest group” – patients who presented with dyspnoea, chest pain, cough and/or syncope
- “generalized body pain/weakness group” – patients who presented with generalized body pain and/or weakness
- “psychiatric group” –patients who presented with psychosis, aggression, hallucinations and/or having taken a drug overdose

Patients who required immediate resuscitation or who were pregnant were not considered for inclusion.

**Fig. 1:**
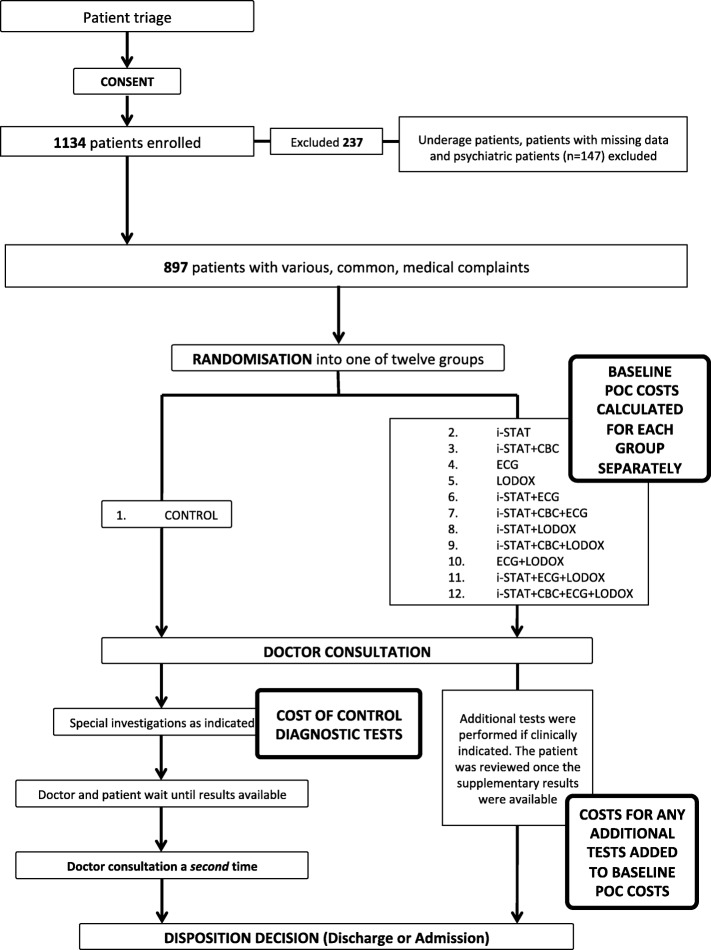
The POC intervention workflows compared to the normal ED patient workflow

Figure 1 demonstrates the methodology followed in the study, showing the normal ED pathway compared to the eleven POC pathways utilised during the study period.

Block randomisation was done prior to study commencement using www.randomizer.org – an online randomisation tool. Randomisation was independent of the nature of the patient presentation. Symptom categories were represented equally in each POC block and all twelve test pathways were assigned to each of the above symptoms groups (Fig. 1). Based on the block randomisation, data collection sheet sets were placed upside-down in the order generated. After the patient signed consent, either the research doctor or the research assistant took the next data collection sheet in the order supplied.

The patients were randomised to receive either the normal ED workflow pathway (i.e. the control) or one of the other eleven intervention POC pathways with various combinations of one, two, three or four POC tests (see Fig. 1). This was done in order to ascertain whether any particular individual POC test or if certain combinations of POC tests could provide the most benefit.

In the control pathway, after triage, consent and randomisation, a doctor evaluated the patient. If diagnostic tests were required, they were ordered as indicated. All blood tests were performed according to standard procedures in the on-site hospital laboratory and if the patient required a blood gas analysis, the doctor would perform this on one of two blood gas analysers available in the ED (Cobas B 221 POC system, Roche Diagnostics or ABL800 Flex, Radiometer). X-rays were performed in the radiology department and the ED staff performed ECGs as required.

The doctor would review the patient a second time once the results of the diagnostic tests were available. This was followed by the disposition decision.

In the enhanced, intervention POC pathways, if the doctor deemed additional investigations over and above the POC tests necessary, those tests were then performed according to the ED standard procedures. Once the additional results were subsequently available, the patients were reviewed.

Patients were not subjected to any form of diagnostic investigation that they would not most likely have received by following the control pathway for each particular symptom group. The main difference between the control workflow pathway and the enhanced, intervention POC workflow permutations was that the tests were performed in the ED at the so-called point-of-care *prior* to the patient seeing the doctor for the first time.

Patient throughput time in the ED consists of administrative time and treatment time [10]. Table 1 contains the definitions, possible confounders and solutions employed in this study to overcome them in order to accurately evaluate the effects of the POC tests on patient time in the ED and therefore the impact on the cost-effectiveness.

**Table 1:** Time frame definitions in the ED

Administrative time	Treatment time
Time from patient arrival to doctor evaluation	Time from doctor evaluation to disposition decision
All patients go through the same process of opening a file and registering on the hospital system in our ED.	After a disposition decision is made, the patient may or may not timeously leave the ED.
The administrative process can be substantially longer on some days than on other days. This would change the wait-times for the patients prior to them presenting to the doctor and would confound the time measurements overall and therefore impact on the cost analysis.	Exit block may lead to a delay in the patient leaving the ED even if a timeous disposition decision is made.
Only treatment time was evaluated.	The disposition decision time was utilised.

### POC tests

The POC equivalents of commonly used special investigations in the ED were chosen – details are provided in Table 2. The POC testing was performed in a private cubicle where the LODOX® (LOw-DOse X-ray) machine was located within the ED. All other testing was done as per standard procedure in the ED. Details of the direct cost comparisons of the diagnostic tests and their POC equivalents are depicted in Table 2.

**Table 2:** POC tests employed and cost comparisons between the control pathway tests and their POC equivalents

*Abbott Point-of-Care i-STAT® System*			
The i-STAT System utilises single-use i-STAT test cartridges (i-STAT, Abbott Point of Care, Princeton, NJ, USA) with a handheld POC blood analyser. The CHEM8+ (sodium, potassium, chloride, total carbon dioxide, ionised calcium, glucose, urea, creatinine, haematocrit, haemoglobin and anion gap) and CG4+ (Lactate; pH; partial pressure carbon dioxide (PCO_2_); partial pressure of oxygen (PO_2_); total carbon dioxide; bicarbonate; base excess and oxygen saturation) were performed on venous blood specimens.			
*Abbott CEL-DYN Emerald 22 benchtop haematology system*			
The CEL-DYN Emerald 22 benchtop haematology system was used. It is capable of providing a POC Complete/Full Blood Count as well as a white blood cell differential count.			
*ECG*			
Philips Pagewriter TC30 ECG machines were utilised to obtain the ECGs. All patients randomised to receive an ECG received a standard 12-lead ECG as well as a right-sided (V1R-V6R) and posterior (V7-V9) ECG. The cost of the ECG was the same in both the control and the intervention groups.			
*LODOX®*			
A Lodox Xmplar-dr was used by a radiographer to perform the LODOX® (LOw-DOse X-ray) radiographs (chest and abdomen, antero-posterior and lateral). The radiation exposure was approximately 339uGy per patient versus a standard chest and abdomen radiograph of approximately 5200uGy [11]. LODOX® is the radiological equivalent of a POC test as it can provide a full body X-ray within 19 s without the patient leaving the ED. Its utility in trauma patients is reasonably well-known but its use as a diagnostic tool for non-trauma patients in the ED has not been evaluated previously [12].			
*Diagnostic tests*	*Cost*	*Point-of-Care equivalent*	*Cost*
Complete Blood Count	4.57	CBC (CEL-DYN Emerald 22)	2.33
Urea, Creatinine, Electrolytes Blood Gas	17.89	i-STAT Chem8 CG4+	27.61
X-ray	121.58	LODOX®	104.17
TOTAL	$ 144.04^a^	TOTAL	$ 134.11^a^

$ Costs shown in US dollars for each individual test

*CBC* Complete Blood Count, *ECG* electrocardiogram, *i-STAT* i-STAT POC tests, *LODOX*® Low-dose x-ray

^a^The direct comparison of net costs for testing between the groups if the costs of the tests alone are shown in isolation at face value i.e. what the cost would be for a patient who received all the standard diagnostic tests compared to a patient who received all the POC tests. It would cost $9.93 less to have all the POC tests. This does not include the costs of other tests that might be ordered (e.g. serum amylase or lipase tests)

### POC costs

Costs for the POC blood tests were obtained from the supplier. Capital and maintenance costs of equipment were included in the prices of all tests whether POC or control diagnostic tests therefore no indirect costs were added. Discounting was not applied. Prices for the control pathway investigations were obtained from the hospital laboratory (blood tests) and radiology department (X-rays/LODOX®). The cost of the ECG was equivalent in both pathways.

When comparing the costs of the intervention permutations to the control group, the costs were calculated as follows:

#### Control group

All tests as ordered by the doctors were included e.g. if the doctor ordered an ECG and a blood gas, only the costs of those two tests were included for that particular patient.

#### Intervention POC permutation groups

The costs of the POC tests specific to the group PLUS any additional tests that were ordered by the doctors were included in the total cost e.g. If the doctor ordered an x-ray for a patient who was in the i-STAT + CBC group, the cost of the x-ray was then added to the total cost for that patient.

### Staffing costs

The cost calculations were performed as per Schilling’s recommendation [5]. Staffing was considered as evenly distributed throughout the year and calculated using doctor and nursing costs only. Using this method, the cost of one minute of ED staffing was calculated to be US$5.37, which is equivalent to US$0.75 per patient per minute in our ED.

### Sample size calculation

The sample size estimation was based on the determination of the effect of workflow pathways within each symptom group that were initially analysed. This required a two-way Analysis of Variance (ANOVA). Based on the detection of at least a medium effect size (f = 0.25 or a 20% difference in times between groups) with 80% power at the 5% significance level, a sample size of 864 patients was required.

### Outcomes

Treatment time was the main outcome measure for assessing the effectiveness of POC interventions. A difference in treatment time of 20% was considered to be clinically significant – this is higher than that utilised in previous studies (9–18%) [2, 9].

The main outcome measure for the cost-effectiveness of upfront, POC tests was the incremental cost effectiveness ratio (ICER).

The ICER was expressed as

$$ \mathrm{ICER}=\mathrm{C}1-\mathrm{C}2/\mathrm{E}1-\mathrm{E}2 $$

where C1 and E1 are the cost and effect (time) in the intervention group and C2 and E2 are the cost and effect in the control group [13].

### Statistical analysis

A cost-effectiveness plane was constructed by plotting the effects on the horizontal axis and costs on the vertical axis. Further analysis utilised a non-parametric bootstrapping model. This model used the observed data for each permutation which was inserted into an excel template supplied by Barton et al [14]. For each bootstrap sample, the mean incremental costs and effects were calculated and repeated 1000 times. Incremental cost-effectiveness acceptability curves were then calculated from the bootstrap data across a range of increasing potentially acceptable costs. This analysis excluded the effects of potential cost-saving related to staffing expenses.

Data analysis was carried out using SAS (version 9.4 for Windows). The 5% significance level was used for all statistical analyses.

## Results

There were 1134 patients enrolled in the trial. Consecutive patients were included during the patient enrolment periods – there was no patient selection. Five patients refused to participate in the study. After exclusions, 1044 patients were randomised. Figure 1 summarises the patient flow.

During data collection for the primary study, the outcomes in the “psychiatric group” (*n* = 147) were found to be very different from the other three symptom groups in an interim analysis. The psychiatric patients were seen almost immediately in most cases based on their “orange” triage scores and commonly only needed a single investigation viz. a blood gas analysis. From an ED-throughput perspective it was already functioning optimally and the extra testing was not required. Their data was therefore excluded as it would have skewed the results from both a time- and cost perspective. Therefore, 897 patients were included in the analysis.

Ten patients presented to the ED on more than one occasion during the study period and agreed to be enrolled a second time. They were treated no differently from patients who were seen for the first time. They all signed a new consent form and were randomised yet again. Their inclusion was therefore unlikely to have influenced the study outcomes.

### Patient characteristics

A comparison of patient characteristics based on workflow allocation is tabulated in Table 3. There were no significant differences in age, triage category or disposition between the patients enrolled into the control or POC intervention groups.

**Table 3:** Patient characteristics based on the twelve workflow allocations

	CONTROL	i-STAT	i-STAT CBC	ECG	LODOX	i-STAT ECG	i-STAT CBC ECG	i-STAT LODOX	i-STAT CBC LODOX	ECG LODOX	i-STAT ECG LODOX	i-STAT CBC ECG LODOX	*p*-value for between group test
N	75	75	74	77	75	74	76	74	77	73	74	73	
Age median (IQR)	45.7 (34.2; 61.7)	45.2 (31.6; 59.3)	44.2 (33.3; 68.0)	44 (33.9; 61.0)	41.6 (33.0; 55.5)	41.9 (33.3; 56.6)	40.7 (27.3; 60.9)	42.1 (29.9; 60.8)	37.1 (30.0; 55.1)	44.9 (35.0; 60.5)	39.5 (30.0; 61.3)	41.3 (31.5; 55.6)	0.65
Sex: Males (%)	30 (40.0)	30 (40.0)	29 (39.2)	36 (46.8)	30 (40.0)	27 (36.5)	35 (46.1)	18 (32.4)	28 (36.4)	31 (42.5)	42 (56.8)	37 (50.7)	0.11
Triage category													0.30
N (%)													
Orange^c^	22 (29.3)	18 (24.0)	11 (15.1)	22 (28.6)	15 20.0)	16 (21.6)	24 (31.6)	18 (24.3)	22 (28.6)	14 (19.2)	18 (24.3)	21 (28.8)	
Yellow^c^	52 (69.3)	54 (72.0)	59 (80.8)	52 (67.5)	60 (80.0)	57 (77.0)	50 (65.8)	54 (73.0)	55 (71.4)	59 (80.8)	56 (75.7)	48 (65.8)	
Green^c^	1 (1.3)	3 (4.0)	3 (4.1)	3 (3.9)	0 (0.0)	1 (1.4)	2 (2.6)	2 (2.7)	0 (0.0)	0 (0.0)	0 (0.0)	4 (5.5)	
Admitted^a b^ N(%)	32 (42.7)	32 (42.7)	40 (54.1)	34 (44.2)	34 (45.3)	29 (39.2)	42 (56.0)	35 (47.3)	32 (41.6)	39 (53.4)	40 (54.1)	39 (53.4)	0.62
Discharged^a^ N(%)	38 (50.7)	41 (54.7)	33 (44.6)	43 (55.8)	41 (54.7)	42 (56.8)	33 (44.0)	39 (52.7)	44 (57.1)	33 (45.2)	34 (45.9)	34 (46.6)	

*CBC* Complete Blood Count, *ECG* Electrocardiogram, *IQR* inter-quartile range, *i-STAT* i-STAT POC tests, *LODOX*® Low-dose x-ray

^a^8.2% of all patients were referred to another speciality as their disposition plan (i.e. neither admitted nor discharged)

^b^The overall admission rate for this ED is usually 30–35%. This includes all patient presentations e.g. trauma, general surgery, orthopaedics, otorhinolaryngology etc. The medical subgroup of patients typically has a higher admission rate than other patients

^c^Target times for the patients in each triage acuity category are Orange (to be seen within 10 min of ED arrival), Yellow (to be seen within 1 h of ED arrival) and Green (to be seen within 4 h of ED arrival)

### Treatment times

A 20% reduction in treatment time was exceeded by all POC workflow permutations, except ECG alone and LODOX® alone groups.

With regards to disposition decision, there were no significant differences in treatment times between patients who were ultimately admitted or discharged within particular workflows, or between admission and discharge within particular symptom groups (*p* = 0.091).

### Time taken for POC testing and patient waiting times

The patient waiting time to see a doctor after arrival in the ED was on average between 57 and 152 min. It took between 4 and 23 min to obtain the results of the POC tests. This included the time taken for phlebotomy, specimen processing and results printing for the i-STAT and CBC permutations. The blood tests could generally be performed concurrently, however, the LODOX® and ECGs had to be performed sequentially.

### Investigation utilisation in the control pathway

There were 78.7% (59/75) patients in the control group who had blood tests and/or a blood gas analysis. Twenty-four per cent (36/75) had a blood gas analysis only. X-rays were performed in 58.7% (44/75) of patients and 64% (48/75) had ECGs performed.

### Costs of investigations

Table 2 lists the costs for the individual investigations. Overall, POC equivalent tests cost US $9.93 less than the standard control investigations if all the tests were performed in a patient.

The time-saving and costs for each workflow is presented in Table 4.

**Table 4:** Costs and time-saving analysis ranked according to net additional cost per patient

	Total Average Group Cost^a^ (US$ pp)	Difference between costs of POC tests and control (US$ pp)	Time Saved – Difference between control group time and POC group time (min)	Staffing costs saved (US$ pp)	ICER - Incremental Cost Effectiveness Ratio (US$ / min)	Net additional cost per patient in POC group (US$ pp)
CONTROL	81.86	–	–	–	–	–
i-STAT + CBC	82.86	1.00	31	23.21	0.03	−22.21
ECG ONLY	75.96	−5.90	9	6.74	−0.65	−12.63
i-STAT + CBC + ECG	90.73	8.87	26	19.47	0.34	−10.60
i-STAT	92.59	10.73	21	15.72	0.51	−4.99
i-STAT + ECG	95.36	13.50	21	15.72	0.64	−2.22
ECG + LODOX	127.48	45.62	25	18.72	1.82	26.90
i-STAT + ECG + LODOX	143.11	61.25	32	23.96	1.91	37.29
ALL POC TESTS	144.10	62.24	31	23.21	2.01	39.03
i-STAT + LODOX	142.56	60.70	25	18.72	2.43	41.98
i-STAT + CBC + LODOX	146.74	64.88	27	20.22	2.40	44.67
LODOX ONLY	142.09	60.23	9	6.74	6.69	53.49

$ Costs shown in US dollars for each permutation

A negative number indicates a lower cost with the POC test permutation than traditional diagnostic testing

*CBC* Complete Blood Count, *ECG* electrocardiogram, *ICER* incremental cost effectiveness ratio, *i-STAT* i-STAT POC tests, *LODOX*® Low-dose x-ray, *pp* per patient

^a^These are the average total actual costs that were incurred for the patients in each permutation. In the Control group, the only tests that were included were those selected by the doctors as they saw fit. In other groups, the average costs appear to be higher than would be expected as extra diagnostic tests may have been performed in those groups over and above those which were assigned (e.g. additional blood tests such as serum amylase or lipase). This principle extends across each of the groups

### Cost effectiveness analysis

**Fig. 2:**
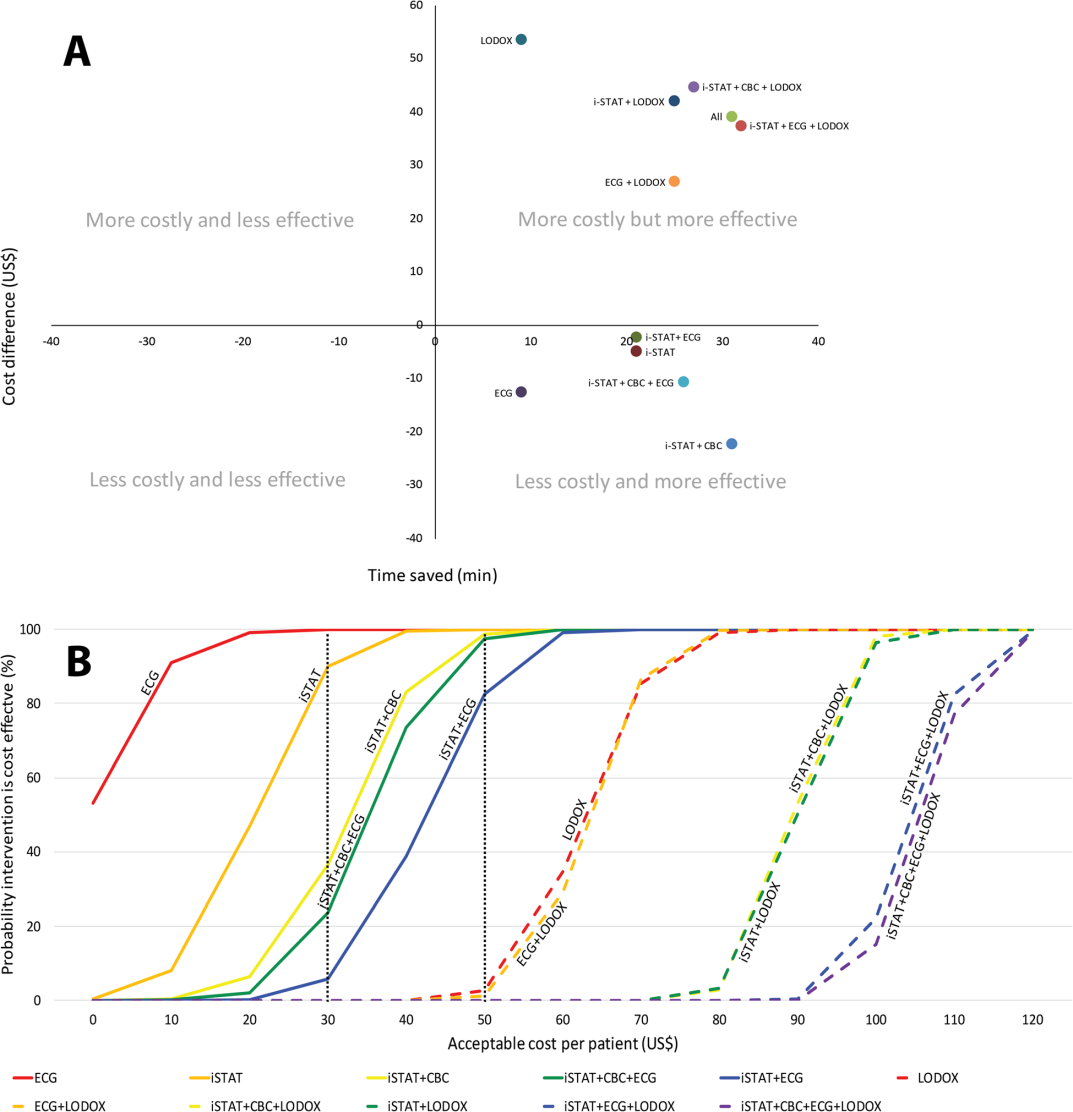
**a** Cost Effectiveness Plane. Permutations in the south-east quadrant were less costly and more effective (also referred to as *dominant*) [13]. Permutations in the north-east quadrant were still more effective but were also costlier. **b** Cost-Effectiveness Acceptability Curve. Cost-effectiveness acceptability curves for each of the permutations. The proportion of the bootstrap datapoints achieving cost-effectiveness at each increment of potentially acceptable cost is shown. Permutations which included LODOX® are shown with dashed lines. The dotted lines represent two potential willingness-to-pay thresholds. For example, at US$50, virtually all the non-LODOX® permutations have a high probability of being cost-effective. On the other hand, at a willingness-to-pay threshold of US$30, only the iSTAT and the ECG permutations have a high probability of being cost-effective. This graph allows the funder to weigh the relative cost of each of the permutations against their known effectiveness. CBC Complete Blood Count, ECG electrocardiogram, i-STAT i-STAT POC tests, LODOX® Low-dose x-ray

Figure 2 exhibits the Cost Effectiveness Plane (2A), which is a graphical representation of the cost effectiveness analysis as well as the cost-effectiveness acceptability curve (2B).

LODOX®-containing permutations (dashed lines) and non-LODOX®-containing pathways (solid lines) are demonstrated using different values for funder willingness-to-pay (**λ**). Non-LODOX® permutations were virtually 100% cost-effective if an additional cost of US$50 per patient was considered acceptable.

## Discussion

Saving time is an ever-present goal in the ED. However, for upfront POC testing to be viable in the ED, the time-saving benefit needs to be weighed against the cost.

### Costs of investigations and cost effectiveness analysis

Variations have been reported with respect to the net cost of POC testing [5, 8, 13]. In Sweden, POC was found to be substantially cheaper than the costs of similar tests performed in a laboratory [5]. In Australia, however, test costs were higher in the POC group [6]. Costs have previously been calculated using the direct differences between that of the POC tests and the laboratory costs without taking the expense of personnel into account [8]. The cost of staffing needs to be taken into account as decreases in test turnaround time could be translated into savings in staffing due to an improved overall processing of the patient from decreased turnaround times as well as quicker diagnosis and ultimately more rapid patient disposition [2, 5, 8].

In our study, direct head-to-head cost comparison between the POC tests compared to standard laboratory and radiological expenses in our study surprisingly showed a saving of US$9.93 if all the tests had been performed on all patients compared to using standard diagnostic tests. This was mainly as a result of the lower cost of the LODOX® compared to the x-ray and the lower cost of the POC CBC compared to the laboratory CBC. Although this comparison of total costs appeared promising, it was necessary to look at the cost-effectiveness of the individual permutations.

When evaluating the cost:benefit ratio for POC testing, it is essential to include the disbursements on staffing. The time a doctor spends with the patient has a cost – if this time can be decreased with POC testing then the cost of the doctor needs to be included in the cost-effectiveness evaluation [2, 5, 8]. When personnel costs and time-saving were both considered, the net additional savings increased further, with the true benefit of certain test combinations being highlighted.

In Fig. 2, it can be seen that all LODOX®-containing permutations fell into the north-east “more costly but more effective” quadrant. X-ray and LODOX® costs formed the bulk of the expenses related to the diagnostic testing combinations making all the LODOX®-containing options more costly. LODOX® therefore added substantially to the cost, without much additional time-saving. Also, only 58.7% of the control group had an x-ray performed while 100% of the participants in the respective LODOX® permutations received an x-ray. This lead to an overall additional comparative cost per patient compared to the control group despite LODOX® being more inexpensive than a standard x-ray. The addition of a LODOX® in a protocolised fashion may need to be re-evaluated and may perhaps be more valuable if introduced only after an admission decision is made. Indiscriminate use of LODOX® on all patients irrespective of whether they require hospital admission would lead to over-testing and unnecessary radiation exposure even if it is relatively low-dose radiation. Some patients also received a formal x-ray in addition to their LODOX® which increased costs and so was a confounder for the LODOX®-containing groups overall.

While the ECG only group was cost-effective because of a direct saving of US$5.90 per patient, the lack of significant time-saving makes it ineffectual to assist with ED throughput.

The most cost-effective combination, which ultimately would save money based on the time-saving, was the i-STAT + CBC permutation. It was second in time-saving to i-STAT + ECG + LODOX® by one minute and equivalent in saving time to the combination where the patients had all the tests performed. The latter two permutations would require additional spending in order for them to be implemented. With one-third of patients having laboratory testing in general in the ED, the i-STAT + CBC option would fulfil the dual purpose of demand and cost-effectiveness [15, 16].

### The impact of staffing costs

Staffing costs play a significant role in the calculation of cost effectiveness. In a Swedish ED, Schilling showed a significantly higher cost saving than in our study. This was largely due to their higher cost of staffing (US$24.08/min versus our US$5.37/min) [5]. A higher staffing cost would mean that time saved using POC testing is ultimately even more economical. The time-saving could potentially also be used to offset staffing costs. There may be an opportunity to reduce staffing levels based on reduced treatment times offered by the POC tests. Optimisation of patient processing means that the costs of staffing need to be taken into consideration [5].

### Value for money – cost-effectiveness acceptability

Permutations in the north-east quadrant of the cost-effectiveness plane were more effective than the control but were also costlier. The determination of whether an intervention offers “good” value for money depends on the funder’s willingness to pay (**λ**) [17]. The range of potential amounts that the funder may be considering are displayed on the x-axis of the cost-effectiveness acceptability curve and can be judged according to the relative probability that an intervention will be cost-effective shown on the y-axis. Figure 2B demonstrates this concept with the majority of the permutations still most likely to be cost-effective at a willingness to pay threshold of US$50, except for those permutations containing LODOX®.

Non-LODOX® permutations were virtually 100% cost-effective if an additional cost of US$50 per patient was considered acceptable by funders.

This model has been used in previous studies on healthcare cost-effectiveness [13, 14, 17]. It is tool that allows decision-makers to balance up costs against non-quantifiable benefits. For example, a reduced waiting time might not have any direct cost implications, but will increase patient satisfaction. A funder might be willing to pay a small additional amount for this but not a large amount. This tool therefore allows the potential funder to better balance the benefits and costs. It also allows the funder to balance quantifiable costs e.g. the decision whether to close a diagnostic laboratory at night in favour of utilising POC tests.

### Waiting times and special investigation use in the ED

Waiting for the results of special investigation such as blood tests, ECGs and radiographs commonly takes two-thirds of a patient’s entire ED length of stay [15]. A substantial amount of time could potentially be saved if these test results were available prior to the doctor’s initial evaluation of the patient. In this study, waiting for results of the intervention POC tests was concurrent with the patients’ wait to be seen by a doctor (minimum waiting time 57 min). This meant that the time taken to perform the POC tests (maximum 23 min) did not cause any significant delays for the patients as it took place during non-valued added time when the patients were waiting to see the doctor.

In Yoon’s analysis of factors increasing length of stay in the ED in Canada, 38.4% of patients had laboratory tests and 44% underwent some form of X-ray imaging. These interventions were associated with longer lengths of stay [15]. In the USA, Gardner et al. found that 33% of patients had laboratory investigations (increasing length of stay by 35.4 to 40.1 min) and 36% had x-rays (increased by 5 to 15 min) [18]. Thirty per cent of discharged patients in a Finnish study by Kankaanpää et al. needed laboratory testing [19]. The laboratory usage in our control group of 30% (excluding patients who had blood gas analyses alone) is similar to the utilisation in these other EDs. The x-ray utilisation rate was higher, however. This may have been due to the higher admission rate at our hospital of 42.7% (versus 11% in the Gardner study) as all patients admitted to the internal medicine service receive an x-ray.

All patients in an i-STAT-containing subgroup in our study showed a decreased treatment time. Although the performance of a LODOX® scan took only on average four and a half minutes, time-saving was only achieved when it was combined with other POC tests. This was similar to the time-saving gained by the performance of an upfront ECG. Gardner et al. found that ECGs only saved time (2.7 min) in those patients who were admitted but added time in patients who were ultimately discharged [18]. In our study, there was no difference in the number of tests performed regardless of disposition decision i.e. whether a patient was ultimately admitted to the hospital or discharged.

### Standing orders versus upfront POC testing and “over-testing”

In the ED, standing orders have been shown to improve patient throughput by reducing disposition time by up to 16.9% [20]. However, these orders are usually only actioned if the ED is full; have had variable uptake by the nursing staff resulting in both over- and under-testing and have not made use of POC devices [16, 20]. Over-testing is frequently quoted as a danger when standing orders are in place or when POC testing is made easily available. There is, however, no evidence to support this [16, 21–23]. In Retezar’s study evaluating triage standing orders, those patients who received the full gamut of tests had a 16% reduction in their mean treatment times. The hypothesis that upfront, protocolised testing leads to over-testing is nullified by her study findings where 98 % of the patients who did not receive the standing orders went on to receive similar investigations once they were seen by a doctor [24]. The cost of POC usage would therefore be unlikely to be exaggerated compared to standard diagnostic test utilisation. In our study, over-testing was possible in the patients who were ultimately admitted to the internal medicine service. Blood tests are commonly performed as a courtesy for those patients even if the results do not impact on the ED disposition decision. These were extra standard blood tests and not POC tests.

### Other potential cost implications

Besides these direct costs, there is also the potential for further cost-saving that may be possible by reducing admission rates. In Fitzgerald et al’s RATPAC trial, which focussed on patients with chest pain in suspected myocardial infarction, POC testing was associated with higher ED costs but lower general inpatient costs [7].

Other non-fiscal “cost savings” should also be evaluated in future POC cost-effectiveness analyses. Although we did not collect data on the patient experience, we acknowledge that their input would have been useful as part of the overall impact of the intervention. The very low refusal rate may have suggested that patients favoured this system, but no direct data were collected.

The doctors’ perceptions of the effectiveness and appropriateness of the upfront POC testing were evaluated as part of this study. They were strongly supportive of the intervention [25].

Further possible positive effects which need to be quantified include the beneficial knock-on effects of decreases in patient complaints due to excess waiting times, increases in staff satisfaction, and the potential for fewer patients leaving the ED without being seen. This will require future investigation.

### Patient sub-groups that could benefit from upfront POC testing

Although the symptom groups originally included in the study characterised typical categories of undifferentiated patients that present to the ED, interim analysis highlighted that the “psychiatric group” was already functioning optimally based on their high acuity triage scores as well as the limited special investigations that they required for safe patient disposition. The use of upfront POC testing in this group of patients would therefore have no time- or cost benefit. Upfront testing appeared to be most appropriate for the undifferentiated medical patient and the ultimate cost-effectiveness in any ED would depend on the case mix presenting to that ED.

### Hospital admission rates and patient acuity

There was no difference between the patients who were admitted to the hospital (sicker patients) compared to those who were discharged from the ED (less ill patients). Both groups of patients benefited from the upfront testing. The overall percentage of patients admitted from the ED was higher than the usual admission rate of the ED of 30–35% (Table 3). These higher admission rates are likely related to the fact that only medical patients were included who, in general, are more ill than the non-medical patient population. They are also the patient group that would potentially benefit most from upfront POC testing. Therefore, it is unlikely that the high level of significant illness was an important source of bias in this study. There was a range of triage categories within each group (not significantly different) that further suggests that this was not an important bias. Furthermore, upfront POC testing is not suggested to be used in *all* patients presenting to the ED. Clearly some patients would not benefit (e.g. *minor* orthopaedic injuries), but it would be best applied to patients with undifferentiated medical presentations. This does mean, however, that EDs that see very few sick patients would benefit less from upfront POC testing.

### Limitations

This single-centre study evaluated the impact of POC on the treatment time but there was no data collected nor assessment of the effect on patient outcome and potential adverse effects of universal testing. However, in previous POC studies, there has been no evidence to support the theory of over-testing [16, 21–23]. The patient medical complaints were heterogeneous. Whilst they were common symptoms in our ED, they may not be representative of other EDs. This was notable with regards to the low incidence of acute coronary syndrome-related chest pain. POC troponin was originally included as one of the i-STAT tests used in our study. Although troponin has been shown to be useful for patients with chest pain or suspected acute coronary syndrome in the ED as well as the presence of raised troponin levels having an association with worse short-term clinical outcomes, we ultimately excluded it for the cost-effectiveness analysis as there would be no benefit for our ED population and would have resulted in over-testing [26, 27]. Similarly, the indiscriminate use of D-dimer testing in this heterogenous population without employing pre-test probability scoring could potentially have been harmful and could also have resulted in over-testing. Therefore D-dimer testing was not included in the upfront testing. As the ED doctors were not blinded to which patients received the upfront POC tests, a Hawthorne-type effect was considered, but there was no evidence to support it. However, as the doctors themselves were recording all the times (and not an impartial observer), this could have been a potential source of error. Due to the funding of allied hospital staff being managed separately, staffing costs were calculated using doctor and nursing costs only. The costs related to x-rays and LODOX® were based on the standard prices charged per patient per investigation as opposed to calculations based on the equipment amortisation costs. The different setup costs of a laboratory and of a POC system were also not taken into account. The performance of “admission tests” for the internal medicine service may have also confounded the diagnostic test utilisation. This may have lead to duplication of tests if the patient was admitted.

## Conclusion

POC testing in the ED was more cost-effective, in certain combinations, than standard diagnostic tests when utilised upfront for patients with undifferentiated common medical complaints in non-resuscitation triage categories. The most economical POC test combination was i-STAT + CBC, which not only saved time, but, also saved the most money per patient. Besides these direct costs, there is also the potential for further cost-saving that may be possible by reducing hospital admission rates as well as the other non-fiscal “cost savings”. These should be evaluated in future POC cost-effectiveness analyses.

## Data Availability

The datasets used and/or analysed during the current study are available from the corresponding author on reasonable request.

## Abbreviations

ANOVA: Analysis of Variance
CBC: Complete Blood Count
CG4+: i-STAT test which includes Lactate; pH; partial pressure carbon dioxide (PCO_2_); partial pressure of oxygen (PO_2_); total carbon dioxide; bicarbonate; base excess and oxygen saturation
CHEM8+: i-STAT test which includes sodium, potassium, chloride, total carbon dioxide, ionised calcium, glucose, urea, creatinine, haematocrit, haemoglobin and anion gap
ECG: Electrocardiogram
ED: Emergency Department
ICER: incremental cost effectiveness ratio
IQR: inter-quartile range
i-STAT: i-STAT system point-of-care tests
λ: funder willingness-to-pay
LODOX®: Low-dose x-ray
POC: Point-of-Care
pp: per patient
